# Topology is the Principal Determinant in the Folding of a Complex All-alpha Greek Key Death Domain from Human FADD

**DOI:** 10.1016/j.jmb.2009.04.004

**Published:** 2009-06-05

**Authors:** Annette Steward, Gary S. McDowell, Jane Clarke

**Affiliations:** University of Cambridge Department of Chemistry, MRC Centre for Protein Engineering, Lensfield Road, Cambridge, CB2 1EW, UK

**Keywords:** DD, death domain, FADD, Fas-associated death domain protein, FADD DD, FADD death domain, Ig, immunoglobulin, B1, bundle 1, B2, bundle 2, TNfn3, third fibronectin type III domain of human tenascin, an Ig-like domain, WT, wild type, TS, transition state, $m_{k_{\text{f}}}$, denaturant dependence of the folding rate constant, $m_{k_{\text{u}}}$, denaturant dependence of the unfolding rate constant, protein folding, phi-value, immunoglobulin, fibronectin type III, helix bundle

## Abstract

In order to elucidate the relative importance of secondary structure and topology in determining folding mechanism, we have carried out a phi-value analysis of the death domain (DD) from human FADD. FADD DD is a 100 amino acid domain consisting of six anti-parallel alpha helices arranged in a Greek key structure. We asked how does the folding of this domain compare with that of (a) other all-alpha-helical proteins and (b) other Greek key proteins? Is the folding pathway determined mainly by secondary structure or is topology the principal determinant? Our Φ-value analysis reveals a striking resemblance to the all-beta Greek key immunoglobulin-like domains. Both fold via diffuse transition states and, importantly, long-range interactions between the four central elements of secondary structure are established in the transition state. The elements of secondary structure that are less tightly associated with the central core are less well packed in both cases. Topology appears to be the dominant factor in determining the pathway of folding in all Greek key domains.

## Introduction

Comparative studies of the folding of homologous domains have proved to be valuable in understanding protein folding.^1,2^ Two principal questions are asked in such studies: the first concerns the pathway; which elements of structure fold early and which fold late? The second is a question of mechanism; it has been suggested that there is a continuum of mechanisms,^3^ from strictly framework, diffusion collision mechanisms characterised by polarised transition states,^4,5^ to pure nucleation condensation mechanisms, characterised by diffuse transition states with strictly concomitant formation of secondary and tertiary structure.^6^ Although in a few protein families, the folding pathway does not appear to be conserved (e.g. protein G and protein L^7^) in many protein families the transition states of homologous proteins are remarkably similar; even where the transition state of one protein is much more structured, the general structural features of the transition state are maintained. This has been observed, for instance, in the immunity proteins Im7 and Im9, which have 60% sequence identity,^8^ and in the immunoglobulin-like (Ig-like) proteins including the Ig domain from human titin, TI I27, and a fibronectin type III domain from human tenascin, TNfn3, which have no significant sequence identity, although they share a common fold.^9–11^ Folding mechanisms within a fold can vary significantly between homologous proteins, even where the transition states have similarly structured regions. In the homeodomain superfamily, for instance, folding mechanisms range from a strict framework/diffusion collision mechanism (engrailed homeodomain) to pure nucleation condensation (hTRF1).^3^ In this case, the mechanism is apparently determined by secondary structure (helical) propensity. Thus, studies of the folding of related proteins suggest that both topology and the nature of the secondary structure content have a role in determining how a protein folds.^12^

We have studied the folding of two protein families extensively. The first is the complex, all-beta Greek key Ig-like fold.^9–11,13–15^ Topology is the dominant factor influencing the folding of Ig-like domains. All these proteins fold via a nucleation condensation process, where key long-range interactions, between residues in the central BCEF strands, form early to nucleate folding. The transition state of these proteins is an expanded form of the native state and local interactions are not involved in determining the folding pathway.

In contrast to the complex Greek key topology of the Ig-like domains, spectrin domains are simple three-helix coiled-coil structures with up-down-up connectivity. Three spectrin domains have been studied in our laboratory (R15, R16 and R17 from chicken brain α-spectrin).^16^^–^^18^ In all three of these proteins, two elements of secondary structure (helices A and C) form and interact early and the third (helix B) forms and packs after the rate-determining transition state. There is some suggestion of a change in mechanism, as is seen in the homeodomain family; R16 folds by a framework-like mechanism, with secondary structure (especially in helix C) preceding tertiary structure formation,^17^ whereas R15 folds by a nucleation condensation mechanism with secondary and tertiary structure forming concomitantly (our unpublished results). R17 shows a mechanism similar to that of R16, but with higher Φ-values and more helical structure in helix A.^18^

**Fig. 1:**
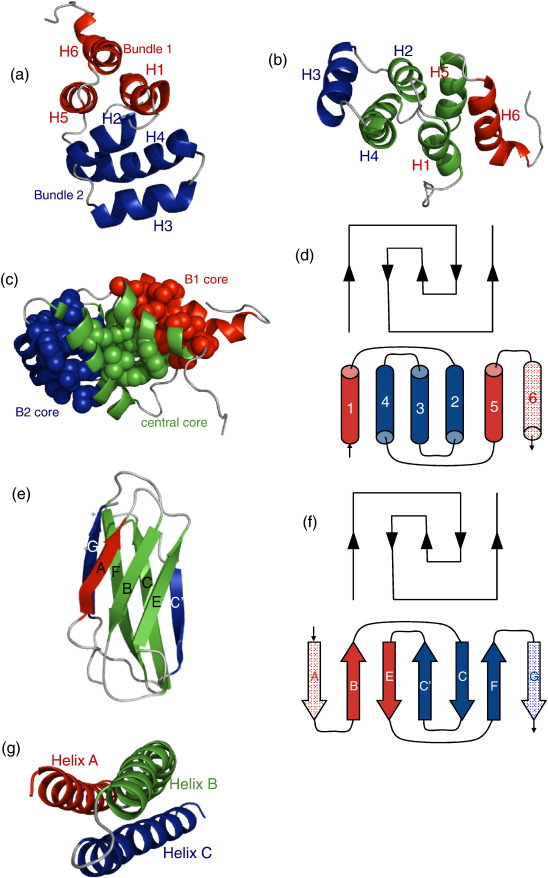
The structure of FADD DD compared to Ig-like and spectrin domains. (a) FADD DD (PDB code ***1E41***) is composed of two three-helix bundles packed orthogonally. Bundle 1 (red) comprises helices 1, 5 and 6; bundle 2 (blue) comprises helices 2, 3 and 4. (b) An alternative view of FADD DD showing that the central region of the protein has two pairs of parallel helices packing against each other (green), H1 and H5 from bundle 1 and H2 and H4 from bundle 2. H3 and H6 are peripheral to the structure and make contacts only within their respective bundles. (c) FADD DD has three structurally distinct cores: two formed by each three-helix bundle (red and blue) and a central core formed by packing of the two pairs of central helices (green). (d) A diagram showing the 2-D topology of FADD DD. Five of the helices display classical Greek key topology.^19^^,^^20^ The helices are coloured to distinguish between bundle 1 (red) and bundle 2 (blue). The helix that is not part of the Greek key motif is shown in pale red. (e) Greek key Ig-like domains (in this case, TNfn3, PDB code ***1ten***) comprise two anti-parallel pairs of strands (one pair from each sheet, shown in green) which pack against each other to form the centre of the single hydrophobic core. Ig-like domains differ in the number and arrangement of peripheral strands (shown in red and blue to distinguish the sheets). (f) A diagram showing the 2-D topology of TNfn3. Five of the strands display classical Greek key topology. The two sheets are coloured in red and blue. Strands that are not part of the Greek key motif are shown in pale colours. (g) Spectrin domains (in this case spectrin R16 from PDB code ***1cun***) are simple three-helix bundles with the same up-down-up arrangement as the bundles of FADD DD.

The principal aim of this study was to compare the folding of a death domain (DD) from human FADD (FADD DD), an all-helical protein with a complex Greek key topology, with the two other classes of protein studied in depth in this laboratory: spectrin domains, simple all-alpha three-helix bundles, and Ig-like domains, all-beta proteins with complex Greek key topology.^19,20^ The death domains provide an opportunity to study the interplay between formation of secondary structure and topology in protein folding. Death domains have six anti-parallel alpha helices, arranged in a Greek key topology, with helices 1, 5 and 6 grouped in an approximately orthogonal position above helices 2, 3 and 4 (Fig. 1).^21^ Human FADD comprises two domains from the DD superfamily, each approximately 100 amino acids; the C-terminal DD is the one studied here.

In the Ig-like domains, two elements of structure, twisted beta sheets, pack together to form the hydrophobic core. Central to this structure is a four-strand motif consisting of two pairs of anti-parallel beta strands, one from each sheet (B-E and C-F, Fig. 1). Similarly, death domains can be thought of as two three-helix bundles, packed together via a central four-helix motif: bundle 1 (B1) is made from the packing of H1, H5 and H6, and forms the B1 core. Bundle 2 is made of contiguous helices H2, H3 and H4, which pack to form the B2 core. The central four-helix motif comprises two pairs of parallel helices (H1-H5 and H2-H4) packed together orthogonally, to form the central core. As is shown in Fig. 1, the three cores are clearly separated, so that different faces of H1, H2, H4 and H5 contribute to different cores. The peripheral helices H3 and H6 contribute only to the packing of the bundle cores (B2 and B1, respectively). In contrast, the Ig-like domains have a single hydrophobic core formed by packing of the twisted beta sheets. Thus, FADD DD can be thought of as two spectrin-like, three-helix bundles packed together to form a Greek key structure with central elements forming the central core in a manner that is reminiscent of Ig-like domains.

## Results

### Studies of wild type FADD DD

**Fig. 2:**
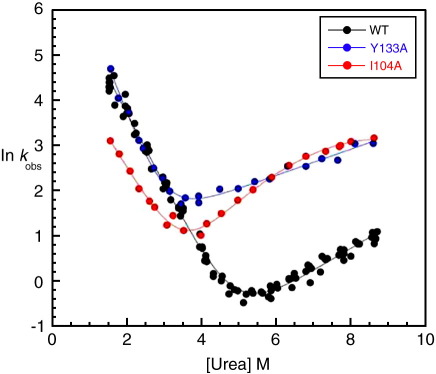
Sample chevron plots for FADD DD. WT (black) and some mutants, such as Y133A (blue), have straight folding and unfolding limbs; other mutants, such as I104A (red), have clearly curved unfolding limbs. This is not a reflection of stability, i.e. the length of the unfolding limb; I104A and Y133A have similar [urea]_50%_ values. Chevron plots for all mutants are included in the Supplementary Data.

The equilibrium stability of wild type (WT) FADD was determined at pH 7.0, 25 °C in urea with unfolding monitored by changes in both intrinsic fluorescence and elipticity at 222 nm. Fluorescence and circular dichroism (CD) data overlaid (data not shown), indicating folding to be a completely reversible, co-operative, two-state transition. Data from five experiments gave an average [D]_50%_ (the concentration of denaturant at which half the molecules are unfolded) of 4.8 ± 0.1 M and a mean *m*-value of 1.4 ± 0.2 kcal mol^− 1^ M^− 1^; this gives a mean free energy of unfolding for WT of 6.7 ± 0.15 kcal mol^− 1^. Kinetic rate constants obtained by fluorescence are in agreement with those obtained by CD. WT FADD DD contains one proline residue, but kinetic data fit well to a single exponential at all concentrations of urea. The chevron plot for WT FADD DD fit well to Eq. (2) (see Materials and Methods) with a linear dependence of both ln*k*_f_ and ln*k*_u_ (where *k*_f_ is the folding rate constant and *k*_u_ is the unfolding rate constant) on the concentration of urea (Fig. 2). No data were analysed below 1.5 M urea, because the refolding rate constants become dependent upon protein concentration. This behaviour has been ascribed to transient aggregation.^22^ Linear extrapolation of chevron data to 0 M urea gives a $k_{\text{f}}^{{\text{H}}_{\text{2}}\text{O}}$ of 960 ± 60 s^- 1^ and a $k_{\text{u}}^{{\text{H}}_{\text{2}}\text{O}}$ of 0.04 ± 0.01 s^- 1^; WT kinetic *m*-values give a β-Tanford (β_T_) value of 0.76 (see Materials and Methods), indicating that the transition state is compact. The free energy of unfolding derived from equilibrium and kinetic data was compared at 2 M urea (to avoid long extrapolation to 0 M urea); the WT kinetic^2M^ stability is 3.5 ± 0.1 kcal mol^- 1^, compared with that from equilibrium^2M^ experiments of 3.9 ± 0.2 kcal mol^- 1^.

### Choice of mutations

To obtain Φ-values for core residues, and residues within elements of secondary structure, 64 mutations were made at 42 positions throughout the six helices: 23 of these were mutations of core residues, and 19 were residues where alanine to glycine “scanning” was performed, to report on the extent of secondary structure formation.^23,24^ Of the 23 core residues, most were non-disruptive replacements by alanine; however, in four cases this mutation was too destabilising and so non-conservative substitutions were made (Leu to Met and Trp to Phe). The residues to which the contacts are deleted and the number of heavy-atom side-chain contacts within 6 Å deleted on mutation are given in Table 1, which illustrates that truncation of core residues either reports mainly on contacts within one of the bundle cores or probes contacts in the central core. Thus, for example, in H4, R140 contacts only residues within B2, whereas L145 only makes long-range contacts with residues in H1 (in B1), through the central core.

### Analysis of mutant data

The change in stability upon mutation (ΔΔ*G*_D-N_) was determined using equilibrium denaturation followed by changes in fluorescence. All equilibrium *m*-values were the same as WT within error, and so ΔΔ*G*_D-N_ was determined using a mean *m*-value (<*m*>) of 1.4 ± 0.21 kcal mol^- 1^ M^- 1^ as:^25^

(1) $$\Delta \Delta G_{\text{D−N}}=\;<m>.\delta {\left[\text{urea}\right]}_{50\%}$$

where δ [urea]_50%_ is the difference in the midpoint of denaturation between WT and mutant protein.

Fitting the kinetic data for FADD DD mutants proved to be complex. All kinetics were measured using intrinsic Trp fluorescence as a probe to allow data to be collected at a low concentration (≤ 1 μM) of protein As for WT, both refolding and unfolding data fit well to a single-exponential equation for all mutants. To avoid the possibility of complication from aggregation events observed in the WT protein, refolding data below 1.5 M urea were not used for fitting the chevron plots. An exception to this rule was made for highly destabilised mutants with a low [urea]_50%_, where the refolding arm was short (W112A, W148F, L161A and L165A). For S144A, all data below 2.5 M urea were omitted from chevron fitting, due to aggregation. All mutant chevron plots had linear folding arms with essentially the same slope (only three had refolding *m*_*k*__f_ values that were significantly different from the mean value of 1.7 M^- 1^).

Some mutant chevron plots had linear unfolding limbs, as was seen in WT. However, many mutants exhibited some downward curvature in the unfolding arm of their chevron plot and for a few mutants this curvature was very significant (Fig. 2). (What curved chevron plots may mean is discussed later). It was therefore not possible to do any global fitting of the data. All chevrons were fit individually: first, the chevrons were fit to an equation with a quadratic term in the unfolding limb only (a “Hammond” fit,^26^ see Eq. (3)); second, each chevron plot was fit individually to an equation describing a linear dependence of both ln*k*_f_ and ln*k*_u_ on the denaturant concentration (linear chevron fit, see Eq. (2)). To fit the kinetic data to the linear chevron fit, data points that were judged to be curving downwards were omitted from the fit. (All chevrons are shown in Supplementary Data Figs. S1 and S2, fit to a linear equation, identifying the points omitted.)

Most importantly, all Φ-values in this work have been determined using refolding data only, to avoid any uncertainty that might arise from fits of the unfolding data. Also, to avoid a long extrapolation to 0 M denaturant^27^ and to eliminate any possible effects of aggregation, all Φ-values were calculated at 2 M urea. We show (Supplementary Data Fig. S 4) that the Φ-values determined using either linear or Hammond fits of the data are essentially identical. Thus, despite the complexity of the analysis, the Φ-values obtained are reproducible. The results of the kinetic analysis are shown in Tables 2 and 3.

### Φ-Value analysis

Φ-Values obtained were either zero or fractional, with no Φ-value of 1 (Tables 2 and 3), indicating no part of the protein is as fully formed in the transition state as it is in the native state. To interpret a Φ-value analysis, it is customary to consider Φ-value patterns, rather than to try to interpret individual Φ-values. This allows one to determine which regions of the protein are fully unfolded, partially folded or fully folded in the transition state.^28^ The Φ-values obtained are thus generally classified into low, medium and high classes, with the boundaries chosen to reflect the overall Φ-value distribution (e.g. see Ref. 29): in our case, the cut-offs used are: low, Φ ≤ 0.15; medium, Φ = 0.16–0.5; high, Φ ≥ 0.51.

We will consider the structure of the transition state of FADD DD in terms of formation of the three structural cores, i.e. formation of the two three-helix bundles B1 and B2, and the formation of the central core (the four-helix motif). (A detailed analysis of each helix is given in the Supplementary Data). The Φ-values were mapped on the native structure, to allow the patterns of Φ-values to be interpreted.

#### Formation of B1 (formed by H1, H5 and H6): (Fig. 3)

Formation of the B1 core was probed by six mutations: **H1**, V103A; **H5**, V162A, L165A; and **H6**, V173A, L176A, V177A. The extent of helix formation was probed by six Ala to Gly mutations: **H1**, A98G, A102G; **H5**, A166G, A167G; and **H6,** A175G, A178G.

All three helices are partially folded but the B1 core can be considered as only very weakly structured. Only H5 and H6 make contact through the B1 core, through interactions between L165 at the C-terminus of H5 and V173 at the N-terminus of H6. H1 is only connected to the B1 core in the native state via V103, but the Φ-value of V103A is zero, suggesting that H1 does not contribute to the formation of the B1 core in the transition state (TS). (Note, however, that H1 and H5 do interact in the TS, through sidechain contacts on the central core side of the helices).

#### Formation of B2 (formed by H2, H3 and H4): (Fig. 3)

Formation of the B2 core was probed by six mutations: **H2**, W112A; **H3**, I126A, I129A, Y133A; and **H4**, R140A, S144A. The extent of helix formation was probed by 12 Ala to Gly mutations: **H2**, A113G, A114G, A117G; **H3**, A127G, A131G, A132G; and **H4,** A138G, A139G, A142G, A143G, A146G, A150G.

Helix 4 is the most structured region of the entire protein, with high Ala to Gly Φ-values at the N-terminal end, becoming lower towards the C-terminal end. The two B2 core residues in this helix both have high Φ-values; W112 (the only hydrophobic residue from H2 which packs into this core) has a medium Φ-value. All the Φ-values of helix 3 are close to zero; this helix plays no role in the formation of B2 in the TS. Thus, we infer that the B2 core is loosely structured in the TS with H2 packing onto H4.

#### Formation of central core formed by H1, H2, H4 and H5 (Fig. 3)

Formation of the central core was probed by nine mutations: **H1**, F101A, I104A; **H2**, L115M, L119M; **H4**, V141A, L145M, W148F; and **H5**, H160A, L161A. The extent of helix formation was probed by 13 Ala to Gly mutations: **H1**, A98G, A102G; **H2**, A113G, A114G, A117G; **H4,** A138G, A139G, A142G, A143G, A146G, A150G; and **H5**, A166G, S167G. (Note that in native FADD DD, H1 and H5 of the central core run parallel with each other and are packed orthogonally onto the parallel helix pair H2 and H4; Fig. 1).

In the TS, the central core is partly formed, principally through interaction of F101 and I104 in H1, which contact residues from all the other three helices; H5 also contributes significantly to core packing, via H160 and L161 at the N-terminal end. The central core residue in H2, L115, which packs onto residues in both H1 and H5 in the native state, has a medium Φ-value. Notably, although H4 is apparently well structured, the only core residue that contributes structure in the TS is V141 at the extreme N-terminus of H4. It has a high Φ-value, and appears to pin this end of H4 to H1 via an interaction with I104. In the native state, the central core of FADD DD is dominated at one end by W148F from H4, which has a Φ-value of zero. Thus, one end of the central core appears to be largely unstructured, and H4 is essentially attached only via contacts with H2 (via the B2 core).

## Discussion

### There is no evidence for the presence of a refolding intermediate

Curvature or ”roll-over” in the re-folding arm of a chevron is good evidence for the presence of a populated folding intermediate. However, it has been shown that curvature in the refolding arm can indicate the presence of transient aggregation at low concentrations of denaturant, as is observed in FADD DD. Any curvature present due to refolding from an intermediate will be masked by this aggregation. The non-concurrence of equilibrium and kinetic data is another indication of the presence of a refolding intermediate accumulating on the pathway.^30^ Where a Hammond fit is used, the agreement between kinetic and equilibrium free energies is good (Supplementary Data). Moreover, the average kinetic and equilibrium m-values, taken from WT and mutants, are the same within error (1.3 ± 0.1 kcal mol^–1^ M^–1^ and 1.4 ± 0.2 kcal mol^–1^ M^–1^, respectively). Furthermore, an amplitude analysis by CD showed no evidence for a dead-time change in amplitude, which would suggest that no helical structure is formed in the dead-time of the stopped-flow experiments (data not shown). Thus, our experiments suggest that, at least above 1.5 M urea where we analyse the kinetic data, the folding of FADD DD is essentially two-state.

### The transition state for folding in human FADD DD

In the TS of the human FADD DD, all three cores are partially structured. Helices H3 and H6, which are peripheral to the structure, are essentially detached from the protein. H3 is the only element of secondary structure that is completely unstructured, and H6 is only pinned to H5 via a helix-turn-helix interaction mediated through V173. In contrast, all four central elements of structure are already associated in the TS: three helices (H1, H2 and H5) contribute to central core packing, with the fourth helix (H4) pinned into the core at one end. However, H2 and H4 clearly interact with each other via residues that are packed in the B2 core. Thus, in the TS, helices H1, H2, H4 and H5 are apparently aligned in two parallel pairs (H1/H5 and H2/H4) and the pairs are packed against each other through interactions between H2 and the H1/H5 pair. Long-range packing interactions (H1 – H5, H2 – H4 and H2 – H1/H5) have a predominant role in the formation of structure in the central four-helix bundle, and in B2. Only in the formation of the B1 core do short-range side-chain interactions appear to be significant (in the formation of the helix 5-turn-helix 6 motif).

Analysis of the secondary structure propensity of FADD DD (as determined using the program AGADIR^31^) suggests that the level of overall helical propensity is very low; only two regions of the protein, the N-terminal region of H4 and the C-terminal region of H6, show any significant helical propensity (> 10%). Interestingly, neither of these regions is involved in forming the central topology-defining core, although the N-terminal region of H4 appears to be important in the folding of B2.

### Curvature in the unfolding arm suggests consolidation of the central hydrophobic core in a late transition state

Downwards curvature in the unfolding arm can formally be caused by: (i) changes in the ground (native) state;^32^ (ii) movement of the transition state along a broad energy barrier;^33,34^ or (iii) a high-energy intermediate separating two transitions states that switch due to, for example, mutation or an increase in the concentration of denaturant.^35^ (See Refs ^36^ and ^37^ for detailed discussion of different explanations for curvature.) Note that in both models (ii) and (iii), mutation of a residue induces curvature when it is in a region of the protein that is more structured in a late transition state than in an early transition state. Thus, analysis of the chevron plots should, in principle, allow us to infer consolidation of the protein as it crosses the transition state barrier(s).^17,38^

**Fig. 4:**
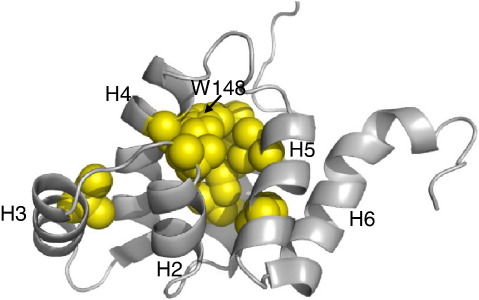
Residues that show strong curvature in unfolding. Apart from a single residue in H3, all the residues that show strong curvature interact in the central core, in particular the residues that surround W148. We infer that the central core consolidates at this end as the protein traverses the TS region of the energy landscape.

There is no evidence for changes in the native state at high concentrations of denaturant. WT and some highly destabilised mutants show no evidence of curvature in the unfolding limbs of the chevron plots, and we see no obvious change in kinetic amplitude. To try to make some qualitative sense of our data, we examined chevron plots that had been fit to a linear equation to determine at which concentration of denaturant deviation from linearity occurred. For most mutants, the unfolding limb was either entirely linear (as in WT) or deviated from linearity only at very high concentrations of urea. A few mutants, however, show very clear deviation from linearity at concentrations of urea below 7 M: F101A and I104A in H1; L119M in H2; I129A in H3; W148F in H4; and L161A and L165A in H5 (Fig. 2, and Supplementary Data Fig. S1). These residues are shown in Fig. 4. Some mutants (L119M, I129A and W148F) have low Φ-values in the “early” transition state (determined at 2 M urea), suggesting that they are not significantly structured, and others (F101A, I104A, L161A and L165A) have medium Φ-values. The significant curvature suggests that the Φ-values of all of these mutants increase at higher concentrations of denaturant. Importantly, nearly all these residues form part of the central hydrophobic core, which is formed by the orthogonal parallel helix pairs: H1/H5 and H2/H4; in particular, they are found towards the C-terminal end of these helices (Fig. 4). In the early transition state structure, described above, the N-terminal part of this core is more structured than the C-terminal part. Thus, the central hydrophobic core appears to consolidate as the protein traverses the transition state barrier. Significantly, buried residues in these same helices, H1, H2, H4 and H5, which pack on the opposite side of these helices into the hydrophobic cores formed by the three helix bundles, do not show similar curvature.

Importantly, the experiments cannot provide information about the order of structure formation. The Φ-values do not give sufficient resolution to determine whether the entire structure folds concomitantly, or if there is an order to the packing of the helices. Future simulations might allow us to detect early events.

### Comparison with other members of the DD superfamily

Clark and co-workers have examined the folding of a number of WT DDs, all of which appear to have complex folding pathways characterised by multiphasic folding and unfolding.^39–42^ Complex folding behaviour has been proposed, and they have suggested that this might be an intrinsic consequence of the complex all-alpha Greek key topology. This would, if true, be in stark contrast to the relatively simple, conserved folding mechanism observed in all-beta Greek key proteins, where populated intermediates are observed only in the more stable Ig-like domains.^43^ Our results for FADD DD demonstrate clearly that these domains do not have intrinsically complex folding pathways. FADD DD folds in a simple two-state manner with no stable kinetic or equilibrium intermediate. Nonetheless, as is true for most of these other DDs, the observed rate constant of folding is somewhat lower than might be expected for such all-alpha proteins with relatively low contact orders.^44^

### Comparison with the all-beta Greek key Ig-like domains

The folding of FADD DD can be compared with that of the all-beta Greek key Ig-like domains studied earlier, including TNfn3,^11,45^ FNfn10,^13^ CAfn2,^14^ and TI I27^9,10^ in our laboratory, and CD2d1.^15^ In the Ig-like domains, tertiary structure is the dominant factor influencing the folding mechanism. These domains exhibit a near-classical nucleation-condensation mechanism where long-range key residues, in the central BCEF strands, interact in the TS to set up the complicated topology; the transition state is an expanded version of the native state, with the folding nucleus involving secondary and tertiary interactions, centred around the structural core. Peripheral regions pack late.

Unlike the Ig-like domains, FADD DD has three discrete hydrophobic cores. The central core is composed of residues from the central helices H1, H2, H4 and H5; the hydrophobic residues interacting in these helices can be regarded as the structural core of this four-helix bundle. The parallel helices H1-H5 and H2-H4 have to be organised relative to each other to form the Greek key topology; this is analogous to the organisation of the anti-parallel beta strands B-E and C-F relative to each other, in the core of the Ig-like domains. In the Ig-like domains, we infer from the Φ-value analysis that the alignment of strands within the sheet and the packing of the two sheets together occurs concomitantly; these are the critical nucleating events for Ig-like domain folding. Although it is not possible to determine the order of events in the folding of the DD from the Φ-value analysis alone, we can show that helices H2 and H4 come together via the core of B2, whereas helices H1 and H5 require the formation of the central core to come together. In both Greek key structures, many long-range, tertiary interactions are involved in formation and stabilisation of the central core; however, local interactions important for helix formation are also involved in the DD. Interestingly, simulations of the TS for folding in the Ig-like protein TNfn3 suggested that the four strands form an “open horseshoe” structure, with all four central strands connected, but with the “ring” of structures incomplete.^45^ This is reminiscent of what we observe in FADD DD with all four central helices in contact, but where the packing of H4 into the central core is only marginal. Another similarity with the Ig-like domains is the observation that the elements of secondary structure most peripheral to the central core are relatively unimportant in formation of the TS structure. In the Ig-like domains, the two beta strands at the N- and C-termini are not involved in forming the structural core, and these fold late. In the DD fold, the N-terminal helix H1 is involved in forming the central core, but H3 and the C-terminal helix H6 are both peripheral to the structure and not involved in packing the central hydrophobic core. The Φ-value analysis suggests that these peripheral helices are either completely unfolded (H3) or attached only loosely (H6) in the TS.

Although FADD DD and TNfn3 have similar stabilities, and appear to have similar folding mechanisms, FADD DD folds significantly faster than TNfn3 (∼ 1000 s^- 1^ compared to 6 s^- 1^). This is not unexpected; FADD DD is an all alpha-helical protein with a significantly lower relative contact order than the all-beta TNfn3, and it has been shown that proteins with low contact orders generally have higher folding rate constants.^44^

### Comparison with other helix bundles

A number of helix-bundle proteins have been studied. FADD DD differs from all of these helical proteins in terms of structure, as three distinct hydrophobic cores can be identified. Here we compare the folding of FADD DD with simple helix bundle proteins. We largely ignore the cytochrome *c* proteins where the haem provides essential stability to the protein,^46^ and the much larger globins, which have more complex hierarchical folding mechanisms.^47–50^

Formation of the central core of FADD involves packing of a four-helix bundle. Several other four-helix bundle proteins have been studied in detail by Φ-value analysis; two members of the ACBP family^51^ and apocytochrome b562^52^ (which are up-down helical bundles) and Im7, the homologous Im9^8^ and the FF domain of HYPA/FBP11^53^ (which have three long helices plus a shorter helix). In all these cases, formation of the TS involves packing of three helices with one helix being essentially unstructured. It has been suggested that the early, obligatory stages in nucleation of folding will be formation of the interactions that are necessary to establish the overall topology.^54–56^ Packing of three helices in a simple four-helix bundle is sufficient to establish the topology. It seems probable that the more complex Greek key topology of the DD requires all four central elements to be assembled, as was found in the Ig-like Greek key domains.

The folding of the two three-helix bundles of FADD DD can be compared with the folding of other three-helix bundle proteins. A number of these have been studied extensively, using Φ-value analysis. In some cases, one well-formed helix is observed in the TS with other elements of structure packed against it (as, for example, in protein A^27,57^), whereas in others, two elements of structure come together with the third helix relatively unstructured (e.g. spectrin domains^17,18^ and peripheral subunit binding domains^58^). The three-helix bundles of FADD DD fall into this second category. Interestingly, as in the spectrin domains, the helices that are in contact are those that are separated in sequence (H1 with H5 and H2 with H4). This suggests that formation of these long-range interactions is the important step for folding these bundles.

## Conclusion

Our Φ-value analysis of the complex all-alpha Greek key FADD DD reveals a striking resemblance to the all-beta Greek key Ig-like domains. Both fold via diffuse transition states and, importantly, long-range interactions between the four central elements of structure are established in the TS. This ensures the topology is established with the correct orientation and pairing of the helices/strands and correct packing of the helix/strand pairs that make up the central hydrophobic core of both proteins. The elements of secondary structure that are less tightly associated with the central core are less well packed in the TS in both cases. Topology appears to be the dominant factor in determining the pathway of folding in all Greek key domains studied to date. However, secondary structure also has a role: whereas in the all-beta Ig-like domains, long-range, tertiary interactions dominate the folding process, in the DD studied here, short-range, local interactions are also important in the folding process, reflecting the helical secondary structure. A recent theoretical study suggests that topology is likely to be the dominant factor in determining folding pathways in all-beta proteins where long-range interactions predominate.^12^ However, in all-alpha proteins, where the majority of stabilising interactions are local, sequence changes are more likely to result in variable folding pathways within a family. The Greek key DDs appear to be an ideal family for the investigation of this theoretical observation.

## Materials and Methods

### Protein expression and purification

A synthetic gene corresponding to residues 93–192 of the DD of human FADD, was obtained from overlapping primers using standard PCR techniques; the synthetic gene was inserted into a modified version of pRSETA (Invitrogen) that encodes an N-terminal histidine tag. Site-directed mutagenesis was performed using a Quik Change kit (Stratagene) and the identity of the mutants was confirmed by DNA sequencing.

Protein expression was carried out in *Escherichia coli* C41(DE3) cells:^59^ transformed cells were grown to an *A*_600_ of 0.4 – 0.6 at 37 °C before induction and growth overnight at 28 °C. The harvested cells were lysed by sonication and the protein purified from the soluble fraction, after centrifugation, by binding to Ni-NTA agarose (Qiagen). The bound protein was cleaved from the resin using thrombin (Sigma) and further purified using a Superdex G75 gel-filtration column (GE Healthcare). The purified protein was stored at 4 °C in 50 mM sodium phosphate, pH 7.0, 150 mM NaCl, 0.2 mM Tris(2-carboxyethyl)-phosphine (TCEP).

### Determination of equilibrium stability

The stability of WT and mutant proteins was determined using urea-induced equilibrium denaturation followed by fluorimetry using a Cary Eclipse spectrometer (Varian); the excitation wavelength was 280 nm, the emission wavelength was 360 nm and the concentration of protein was ≤ 1–2 μM. The stability of the WT protein was also determined by CD using an Applied Photophysics Chirascan circular dichroism spectropolarimeter; data were collected at 222 nm and the concentration of protein was ≤ 5 μM. All experiments were performed at 25 °C in 50 mM sodium phosphate, pH 7.0, 150 mM NaCl, 5 mM DTT. The data were fit to a two-state transition as described.^60,61^

### Kinetics

For WT and mutants, refolding and unfolding were monitored by changes in fluorescence, using an Applied Photophysics SX.18MV stopped-flow fluorimeter. An excitation wavelength of 280 nm was used with a 320 nm cut-off filter; the final concentration of protein was 1–2 μM. WT kinetics were also monitored by CD using an Applied Photophysics Π⁎-180 instrument, with a final maximum concentration of protein of 5 μM. In both cases, the stopped-flow apparatus was maintained at 25(± 0.1) °C. Data collected from 8–12 experiments were averaged and traces were fit to a single-exponential function. Kinetic traces were analysed using Kaleidagraph (Synergy Software).

### Φ-Value analysis

The variation of the logarithm of the observed rate constant with concentration of urea was analysed using Eq. (2); those unfolding rate constants judged to be non-linear were omitted from the fit (see Supplementary Data):

(2) $$\ln\;k_{\text{obs}}=\ln\left(k_{\text{f}}^{{\text{H}}_{\text{2}}\text{O}}\exp\left(-m_{k_{\text{f}}}.[\text{urea}]\right)+k_{\text{u}}^{{\text{H}}_{\text{2}}\text{O}}\exp\left(m_{k_{\text{u}}}.[\text{urea}]\right)\right)$$

where *k*_obs_ is the observed rate constant, $k_{\text{f}}^{{\text{H}}_{\text{2}}\text{O}}$and $k_{\text{u}}^{{\text{H}}_{\text{2}}\text{O}}$ are the folding and unfolding rate constants in 0 M denaturant, respectively, and *m*_*k*__f_ and *m*_*k*__u_ are the gradients of the folding and unfolding arms, respectively.

The variation of the natural logarithm of the observed rate constant with the concentration of urea was also analysed using Eq. (3), which includes a term to account for curvature in the unfolding limb (Hammond fit):

(3) $$\ln k_{\text{obs}}=\ln\left(k_{\text{f}}^{{\text{H}}_{\text{2}}\text{O}}\exp\left(-m_{k_{\text{f}}}.\left[\text{urea}\right]\right)+k_{\text{u}}^{{\text{H}}_{\text{2}}\text{O}}\text{exp}\left(m_{k_{\text{u}}}.[\text{urea}]+m_{k_{u}}^{'}.{\left[\text{urea}\right]}^{2}\right)\right)$$

All Φ-values were calculated at 2 M urea using Eq. (4):

(4) $$\Phi =\frac{\Delta \Delta G_{\text{D−}‡}}{\Delta \Delta G_{\text{D−}N}}$$

where ΔΔ*G*_D-N_ is the equilibrium constant for unfolding determined from equilibrium denaturation experiments and ΔΔ*G*_D-‡_ is the change in the free energy difference between the denatured (D) and transition (‡) states for folding upon mutation, calculated as:

(5) $$\Delta \Delta G_{\text{D−}‡}=RT\;\ln\left(k_{\text{f}}^{\text{wt}}/k_{\text{f}}^{\text{mut}}\right)$$

$k_{\text{f}}^{\text{wt}}$ and $k_{\text{f}}^{\text{mut}}$ are the folding rate constants at 2 M urea for WT and mutant protein, respectively.

The β-Tanford (β_T_) value was calculated using Eq. (6); *m*_*k*__f_ and *m*_*k*__u_ values were derived from chevrons fit to Eq. (2) only:

(6) $$\beta_{\text{T=}}\frac{m_{k\text{f}}}{m_{k\text{f}}+m_{k\text{u}}}$$

## Figures and Tables

**Fig. 3:**
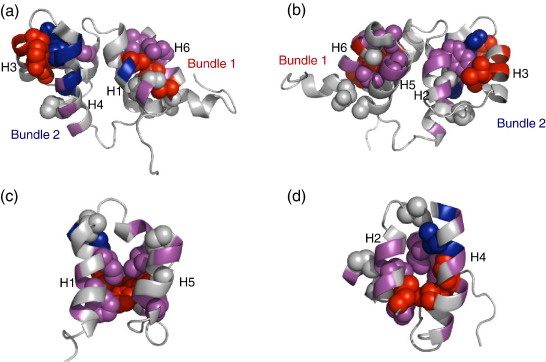
The Φ-values mapped onto the native structure of FADD DD. High Φ-values (≥ 0.51) are shown in blue, medium Φ-values (0.16–0.5) are shown in magenta and low Φ-values (≤ 0.15) are shown in red. The Ala to Gly scanning surface mutations are shown by colouring the ribbon, the buried residues mutated are shown as spheres. (a and b) The Φ-values of the three helix bundles from different faces of the molecule. (c and d) The Φ-values of the central core from different faces. H3 and H6 have been removed to allow a clear view. In c, H6 would be facing the viewer; in (d) H3 would be facing the viewer.

**Table 1:**
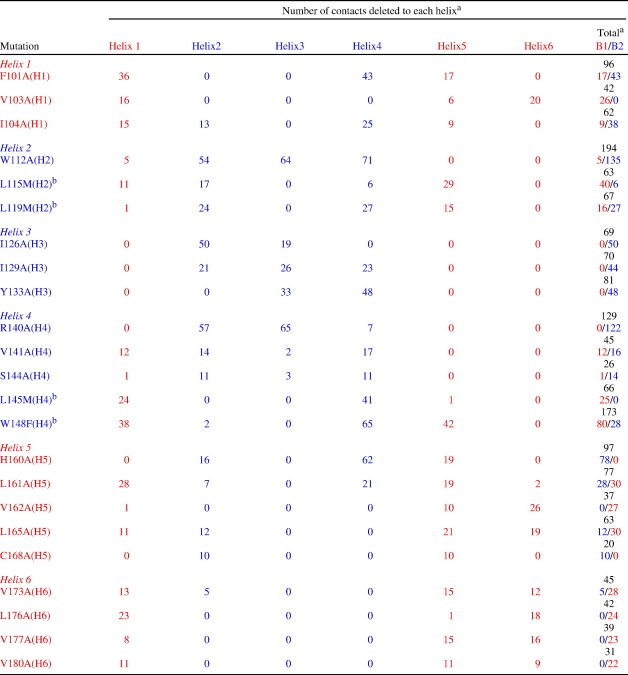
Contacts deleted on mutation of core residues

Data calculated for the FADD DD NMR structure *1e41*,^21^ using the program InsightII (Accelrys Inc.). ^a^ Total contacts made by sidechain (excluding Cβ) to other sidechain heavy atoms within 6 Å is shown in black. Contacts made to other helices within each bundle shown in red for B1 (H1, H5 and H6) and blue for B2 (H2, H3 and H4). Thus, for example, V103 (H1) makes long-range contacts only within B1, whereas I104 (H1) makes the majority of its long-range contacts with residues in B2 (through the central core). ^b^ Note that for non-conservative replacements, these contacts are simply those made by the WT sidechain.

**Table 2:** Folding data for FADD DD core mutants

FADD DD variant	Core probed by mutation	Δ*G*_D-N_ (kcal mol^-1^)	ΔΔ*G*_D-N_ (kcal mol^-1^)	*m*_D-N_ (kcal mol^-1^ M^-1^)	*k*_f_^2M^ (s^-1^)	Φ^2M^
WT		6.7		1.4	40	
*Helix1*						
Phe101Ala	Central	4.2	2.6	1.6	10	0.31
Val103Ala	B1	5.3	1.4	1.2	36	0.04
Ile104Ala	Central	4.3	2.4	1.1	12	0.29

*Helix2*						
Trp112Ala	B2	2.8	3.9	1.4	10	0.18
Leu115Met	Central	5.1	2.4	1.4	22	0.22
Leu119Met	Central	4.3	1.6	1.3	28	0.10

*Helix3*						
Ile126Ala	B2	5.6	1.2	1.2	36	0.05
Ile129Ala	B2	4.5	2.2	1.5	43	–0.02
Tyr133Ala	B2	4.5	2.2	1.1	39	0.01

*Helix4*						
Arg140Ala	B2	7.3	–0.6	1.1	72	0.60
Val141Ala	Central	5.5	1.3	1.3	14	0.51
Ser144Ala	B2	7.4	– 0.7	2.1	114	0.86
Leu145Met	Central	4.5	2.3	1.4	23	0.15
Trp148Phe	Central	3.6	3.1	1.4	55	–0.06

*Helix5*						
His160Ala	Central	7.7	–1.0	1.3	80	0.40
Leu161Ala	Central	2.9	3.9	1.6	4	0.37
Val162Ala	B1	4.9	1.9	1.5	16	0.29
Leu165Ala	B1	2.8	3.9	1.6	7	0.26
Cys168Ala	Central	6.4	0.4	1.4	42	n.d.

*Helix6*						
Val173Ala	B1	5.2	1.1	1.4	29	0.17
Leu176Ala	B1	6.0	0.8	1.3	25	0.37
Val177Ala	B1	4.0	2.7	1.4	24	0.11
Val180Ala	B1	6.8	–0.1	1.2	47	n.d.

For clarity, errors are not shown in the table. The error in free energy measurements is in the range ± 0.1-0.2 kcal mol^- 1^, the error in *k*_f_^2M^ is ± 10%, the error in Φ^2M^ < 0.1. Note that Φ-values cannot be considered reliable if the ΔΔ*G*_D-N_ is low^27^, so Φ-values were not determined where ΔΔ*G*_D-N_ < 0.6 kcal mol^- 1^ (n.d). We note that a condition of Φ-value analysis is that conservative mutations should be made^30^; where this is not possible, for example, Trp to Phe, these results should be interpreted with caution and in the context of the surrounding Φ-values obtained.

**Table 3:** Folding data for FADD DD helix scanning mutants

FADD DD variant	Δ*G*_D-N_ (kcal mol^-1^)	ΔΔ*G*_D-N_ (kcal mol^-1^)	*m*_D-N_ (kcal mol^-1^ M^-1^)	*k*_f_^2 M^ (s^-1^)	Φ^2 M^
*Helix 1*					
Cys98Ala	7.2		1.8	48	
Cys98Gly	6.2	1.0	1.6	26	0.38
Asn102Ala	6.9		1.3	58	
Asn102Gly	6.3	0.7	1.3	33	0.50

*Helix2*					
Arg113Ala	6.5		1.2	52	
Arg113Gly	5.4	1.1	1.1	38	0.17
Arg114Ala	6.4		1.6	46	
Arg114Gly	5.0	1.4	1.6	28	0.20
Arg117Ala	6.3		1.2	47	
Arg117Gly	5.2	1.1	1.1	31	0.23

*Helix3*					
Asp127Ala	7.2		1.5	49	
Asp127Gly	6.2	1.0	1.4	45	0.07
Ser128Ala	7.0		1.1	37	
Ser128Gly	6.6	0.43	1.4	47	n.d.
Asp131Ala	7.5		1.4	44	
Asp131Gly	6.5	1.0	1.4	46	–0.03
Arg132Ala	6.6		1.2	40	
Arg132Gly	5.5	1.1	1.1	43	–0.04

*Helix4*					
Thr138Ala	7.1		1.4	68	
Thr138Gly	6.4	0.7	1.5	35	0.56
Glu139Ala	6.4		1.4	29	
Glu139Gly	5.7	0.7	1.3	16	0.49
Arg142Ala	5.9		1.3	32	
Arg142Gly	4.8	1.1	1.3	12	0.51
Glu143Ala	6.6		1.4	34	
Glu143Gly	4.2	2.4	1.7	10	0.29
Arg146Ala	6.3			30	
Arg146Gly	5.1	1.2	20	20	0.19
Asn150Ala	6.8		2.0	46	
Asn150Gly	6.2	0.6	1.5	32	0.35

*Helix5*					
Arg166Ala	4.6		1.3	22	
Arg166Gly	3.7	0.9	1.6	13	0.30
Ser167Ala	7.6		1.7	69	
Ser167Gly	6.4	1.3	1.7	28	0.42

*Helix6*					
Asp175Ala	6.4		1.4	38	
Asp175Gly	5.4	1.0	1.5	23	0.12
Gln178Ala	6.6		1.2	49	
Gln178Gly	5.6	1.0	1.4	27	0.31

The ΔΔ*G*_D-N_ and Φ-values are determined for the Ala to Gly mutation. For clarity, errors are not shown in the table. The error in free energy measurements is in the range ± 0.1–0.2 kcal mol^-1^; the error in *k*_f_^2M^ is ± 10% and the error in Φ is < 0.1. Note that Φ-values cannot be considered reliable if ΔΔ*G*_D-N_ is low,^27^ so Φ-values were not determined when ΔΔ*G*_D-N_ < 0.6 kcal mol^- 1^ (n.d.).
