# Supplementary material for: Topology is the Principal Determinant in the Folding of a Complex All-alpha Greek Key Death Domain from Human FADD
Source: J Mol Biol. 2009 Jun 5;389(2-3):425–37. doi: 10.1016/j.jmb.2009.04.004 (PMC2724026; doi:10.1016/j.jmb.2009.04.004)
Supplement: Supplementary Material [file mmc1.pdf]

Supplementary Material for:

Topology is the principle determinant in the folding of a complex all-alpha Greek key Death Domain from Human FADD

Annette Steward, Gary McDowell and Jane Clarke

(1) Φ-values in the individual helices

The Φ-values obtained are thus generally classified into low, medium and high classes: in this case the cut-offs used are: low, $\Phi \leq 0.15$; medium, $\Phi = 0.16\text{-}0.5$; high, $\Phi \geq 0.51$.

Helix I has an a-symmetrical pattern of core Φ-values. Most Φ-values, for both core and Ala-Gly scanning mutants are medium indicating that H1 is partially folded in the TS; an exception is V103A which packs into the B1 core which has a Φ-value of zero.

Helix 2 has medium Φ-values for core and secondary structure mutants, except for L119 towards the C-terminus, which has a low core Φ-value (central core).

Helix 3, all Φ-values are zero. This helix is evidently essentially unstructured in the TS.

Helix 4 has an a-symmetrical pattern of core Φ-values. It has high Φ-values at the N-terminus, for both secondary and tertiary structure; towards the C-terminus, the Ala-Gly scanning Φ-values are medium, those of the core residues are low or zero. The helix seems to be more structured at the N-terminus than the C-terminus in the TS.

Helix 5 shows medium Φ-values for all positions. The core Φ-values reduce as they move from the N- to the C-terminus. A Φ-value could not be obtained for C168A at the C-terminus of helix 5 as the $\Delta\Delta G_{D\text{-}N}$ value was below the cut-off of 0.6 kcal mol^{-1}.

Helix 6 shows a mixture of low and medium Φ-values. The Ala-Gly Φ-values suggest that the protein is more helical at the centre of the helix than the ends and the core Φ-values are higher towards the N-terminus than towards the C-terminus. The $\Delta\Delta G_{D\text{-}N}$ was too low for V180A to obtain a Φ-value.

Figure Legends:

Supplementary Figure 1: Chevron plots for all core mutants. The plots for each helix are shown separately. The data in open symbols were excluded from the linear fits.

Supplementary Figure 2: Chevron plots for all surface mutants. The plots for each helix are shown separately. The Ala mutant is shown as a circle and the Gly mutant as a diamond. The same colour is used for mutants of the same residue. The Φ-values were determined by comparing the Ala and Gly mutations at each position. The data in open symbols were excluded from the linear fits.

Supplementary Figure 3: A comparison of free energy of unfolding, $\Delta G_{\text{D-N}}$, calculated from equilibrium and kinetic data at 2 M urea. The dashed line indicates a slope of 1. (a) ΔG_{kin} determined using chevron plots with linear unfolding limbs (points that clearly deviate from a linear fit have been removed, see text and Supplementary Figures 1 & 2). (b) ΔG_{kin} determined using "Hammond" fits, all data points included, solid line is a linear fit of all points, slope = 1.06, R = 0.94. The values are generally in good agreement but the equilibrium value is consistently around 10% higher than the kinetic value where linear fits are used (a). This probably arises from uncertainties in estimating k_u, in particular where there is curvature in the unfolding limbs of the chevron plot. Where a Hammond fit is used the agreement between kinetic and equilibrium free energies is good (b).

Supplementary Figure 4: Comparison of Φ-values from linear and "Hammond" fits of the data. There is no significant difference.

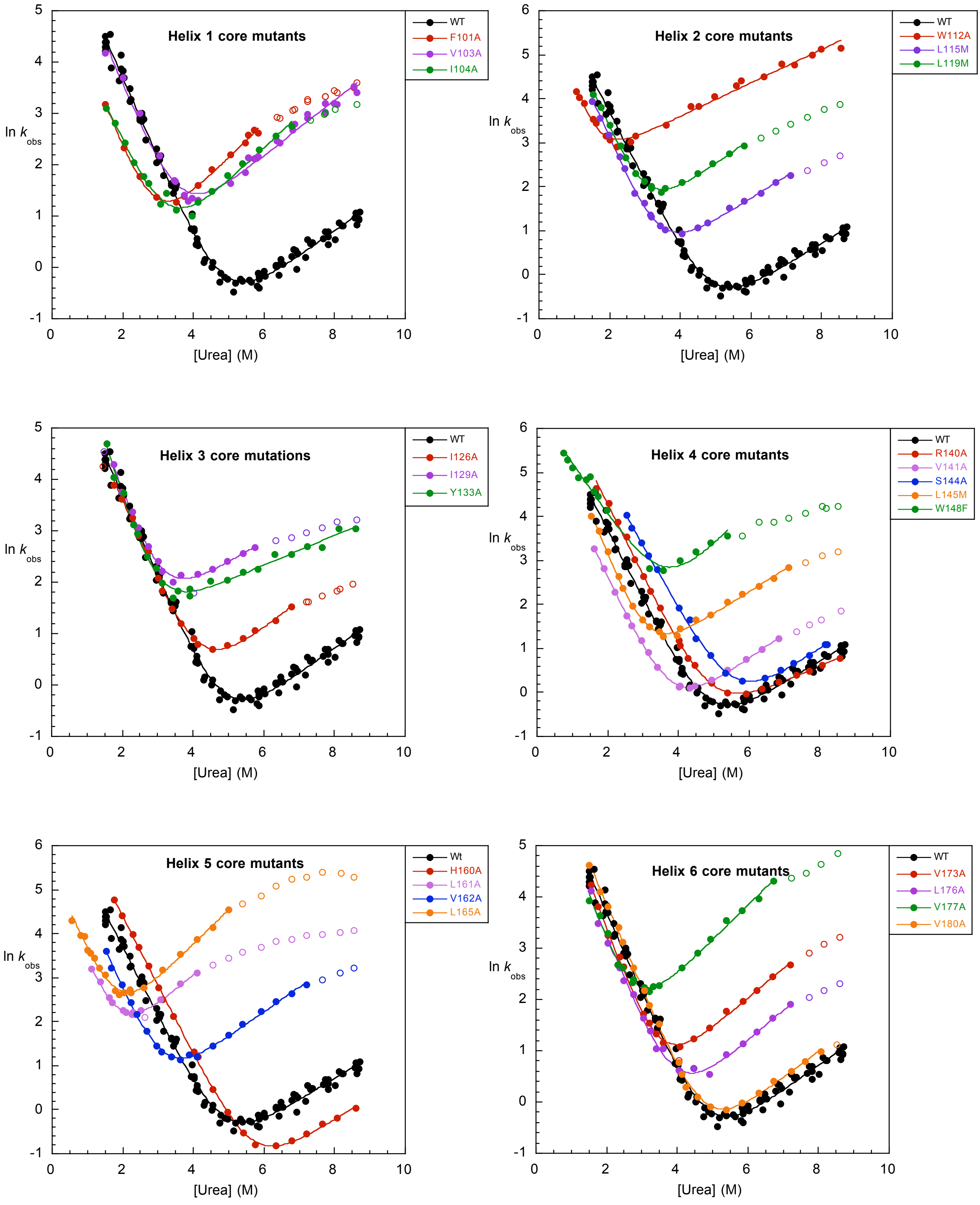

Supplementary Figure 1

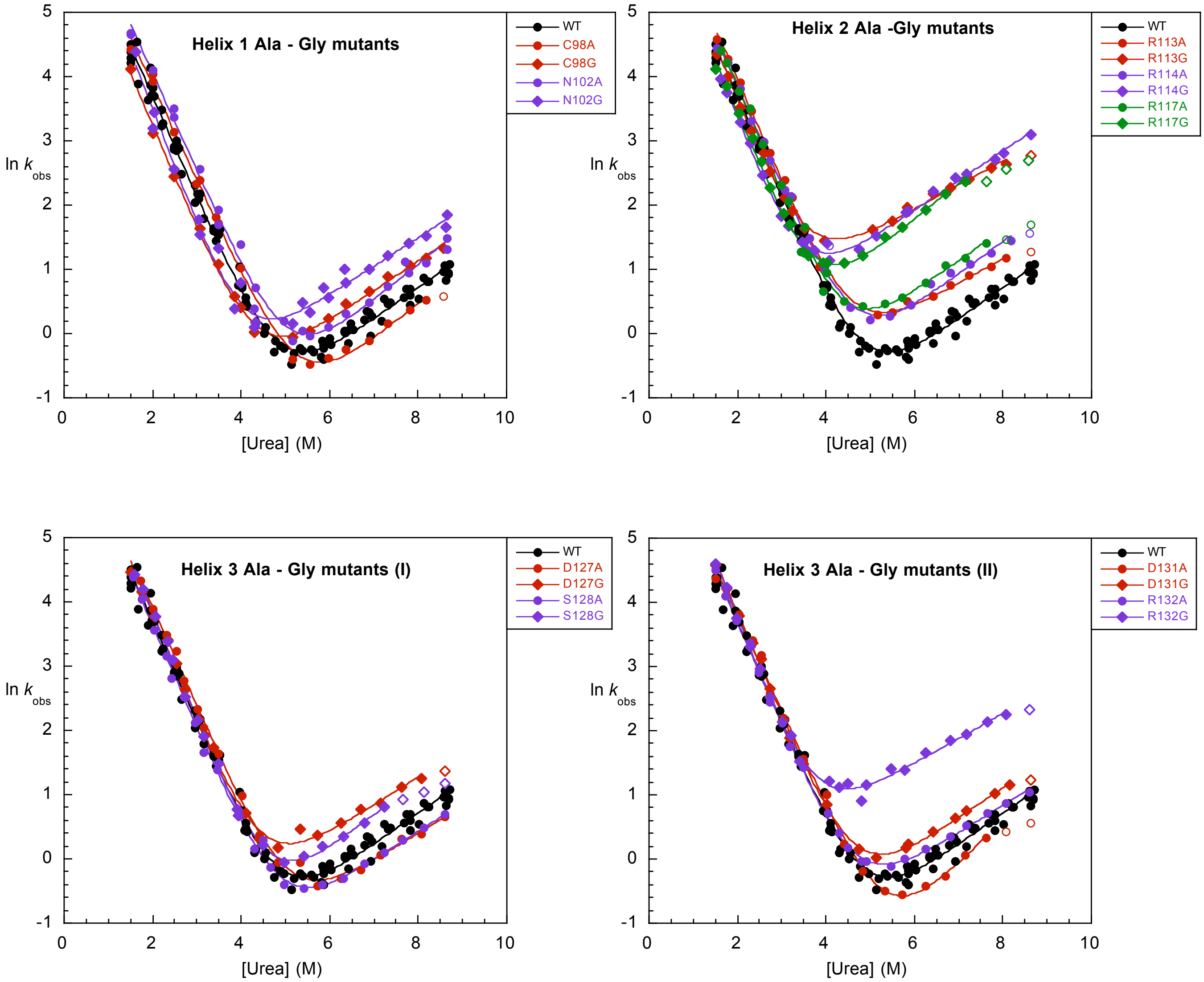

Supplementary Figure 2

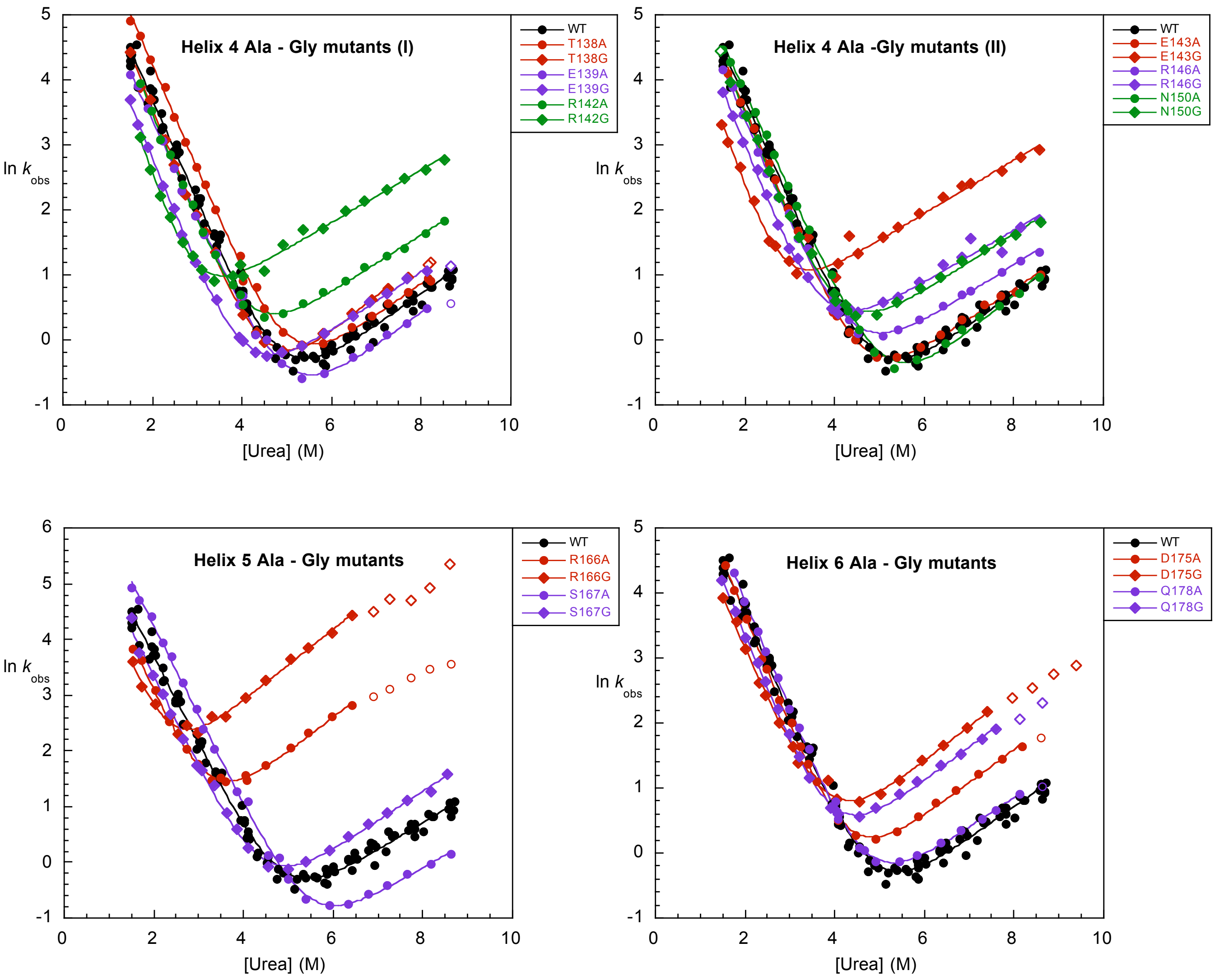

Supplementary Figure 2 contd.

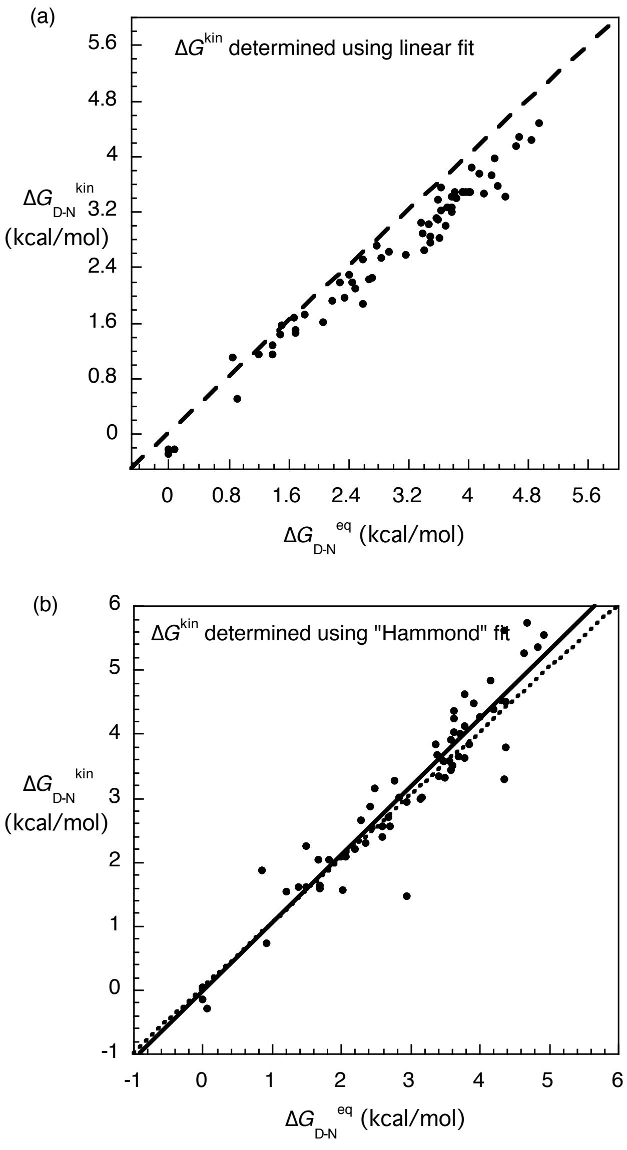

Supplementary Figure 3

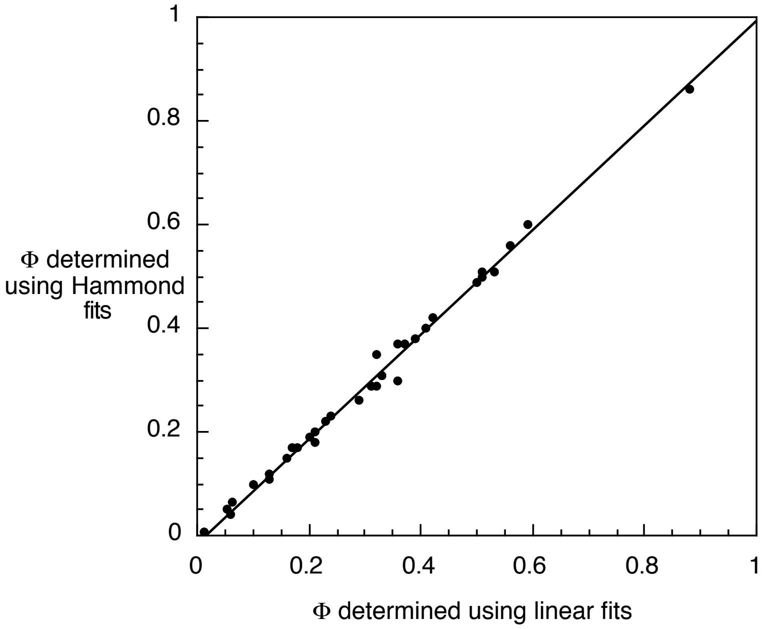

Supplementary Figure 4
